# Influence of Border-Keepers’ Support on Work-Family Enrichment of Preschool Teachers in China: The Mediating Role of Work-Family Boundary Flexibility

**DOI:** 10.3389/fpsyg.2021.752836

**Published:** 2022-02-09

**Authors:** Qian Peng, Chongyan Lian, Limin Zhang

**Affiliations:** ^1^Department of Early Childhood Education, School of Education, South China Normal University, Guangzhou, China; ^2^Meizhou Technician College, Meizhou, China; ^3^Department of Early Childhood Education, School of Education, Guangzhou University, Guangzhou, China

**Keywords:** border keeper’s support, organizational support, family support, preschool teachers in China, work-family enrichment, boundary flexibility

## Abstract

Based on work-family border theory and work-home resource theory (W-HR), this paper examines the impact of border keeper’s support (organizational support and family support) on work-family enrichment and whether or how work-family boundary flexibility mediates the relationship between border keeper’s support and work-family enrichment. A sample of 504 preschool teachers in Guangdong province, China completed questionnaires. The research results show a two-way process of work-family enrichment for preschool teachers in China. Organizational support was directly and significantly correlated with work-to-family enrichment (WFE), and family support was significantly and directly correlated with family-to-work enrichment (FWE). Organizational support had no significant positive predictive effect on work boundary flexibility which has a significant positive predictive effect on WFE. Family support had a significant positive predictive effect on family boundary flexibility which had a significant positive predictive effect on the FWE. In addition, the study found that family boundary flexibility mediates the relationships between family support and FWE whereas work boundary flexibility did not mediate the relationships between organizational support and WFE. The above research results are partly consistent with the existing research, and partly inconsistent, which is related to the profound influence of traditional culture in Chinese society and the current situation of preschool teachers in China. Such findings have important implications for improving the work-family enrichment of preschool teachers.

## Introduction

Work-family border theory is a theory that explains how individuals manage and negotiate the work and family spheres and the borders between them in order to attain balance. Central to this theory is the idea that work and family, constitute different domains which influence each other (Clark, 2000). Since work and home generally differ in purpose and in culture (Clark, 2000), work and family lives may interfere with one another which positive aspect of the work-family interface commonly is referred to as work-family enrichment (Siu et al., 2015). As many scholars have observed, the work-family literature has been dominated by a conflict perspective (Frone, 2003; Powell, 2006; Wayne et al., 2007). Frone et al. (1997) attempted to model the reciprocal (i.e., feedback) relations between work and family life. Given the increasing attention that positive psychology has been garnering today, work-life researchers have started focusing on the positive side of the work-family interface (Carlson et al., 2006; Greenhaus and Powell, 2006). Although numerous constructs have been offered to reflect this positive side of the work-family interface (e.g., enhancement, positive spillover, enrichment, facilitation; Wayne, 2009), one of the most comprehensive frameworks offered to date is offered by Greenhaus and Powell (2006), who define work-family enrichment as the “extent to which experiences in one role improve the quality of life (namely performance and affect) in the other role” (p. 73). Work-family enrichment is one construct representing how work and family benefit each other (Carlson et al., 2006) and a form of synergy in which resources associated with one role enhance or make easier participation in the other role. The resources associated with the work domain may facilitate the performance of family duties and activities, whereas family resources may enhance job performance (Voydanoff, 2005; Grzywacz et al., 2007). Work-family enrichment can occur bidirectionally, meaning that work can provide gains that enhance functioning of the family domain (work-to-family facilitation) or family can provide gains that enhance functioning of the work domain (family-to-work facilitation). Developmental opportunities, such as participation in training and development, are energy resources that promote gains in the work domain that benefit functioning of the family. When these energy resources fostering individual development are successfully exploited by the individual, work-to-family facilitation occurs (Wayne et al., 2007). Carmona-Cobo et al. (2021) proved that daily work-to-family facilitation predicted recovery experiences during off-job time in the evening. Theory of the work-family interface focuses on daily role changes (Ashforth et al., 2000). Through consultation and interaction with important others in the work or family field, individuals can coordinate and perform their roles and responsibilities in the two fields, and finally form a two-dimensional-four-factor model of the work-family interface: work-to-family enrichment (WFE) and family-to-work enrichment (FWE) (Grzywacz et al., 2007). The so-called “important others” here can be called “border keepers.” Since work and family activities are generally carried out with others, border and domain creation and management become an inter-subjective activity in which several sets of actors-border-crossers, border-keepers, and other domain members-negotiate what constitutes the domains and where the borders between them lie. Some domain members who are especially influential in defining the domain and border will be referred to as border-keepers. Common border-keepers at work are supervisors; the most critical border-keepers at home are spouses (Clark, 2000). Employees are border-crossers who make daily transitions between these two settings, often tailoring their focus, their goals, and their interpersonal style to fit each unique demands (Clark, 2000). Border-keepers such as supervisors and spouses have definitions of what constitutes “work” and “family,” and many of them carefully guard the domains and the borders to such a degree that border-crossers do not have flexibility to deal with conflicting demands (Clark, 2000). So border-keepers play an important role in the border-crosser’s ability to manage the domains and borders. Support from “border keepers” is positively correlated with work-family enrichment (McNall et al., 2010, 2014; Bansal and Agarwal, 2020).

Not only is there disagreement between individuals about the borders, including how flexible and permeable they are or should be, there is also disagreement about what constitutes each domain (Clark, 2000; Zhou et al., 2020). Border theory indicates that boundaries between life domains (e.g., work and family) can range from highly segmented to highly integrated (Ashforth et al., 2000). Highly integrated roles can often lead to the blurring of roles, making boundary creation and/or maintenance quite difficult (Lawson et al., 2013). The border between work and family domains has the characteristic of flexibility, if family were willing to take on more responsibilities in the family domain (e.g., sharing housework or picking up children), employees could save more time and energy from the family domain to deal with their work responsibilities. As a result, the family would interfere less with work and the risk of work-family conflict would decrease. Support from border keepers would increase the flexibility of work or family (Frone et al., 1997).

Studies have suggested that a supportive workplace is very important, as it is intrinsically related to various organizational and employees’ positive outcomes, including financial, emotional, instrumental, psychological resources or social-capital support (Voydanoff, 2004; Greenhaus and Powell, 2006) to increase job satisfaction (Li et al., 2015). Supportive family has a substantiated relationship with both increased job satisfaction and decreased family to work conflict (Ford et al., 2007). Some recent studies do suggest that it is an event that border keeper’s support positively affects work-family enrichment (Wattoo et al., 2018), however, very few studies in the past have explained how support actually leads to work-family enrichment due to a lack of empirical studies testing the presence of mediation effects. The direct effects of work-family enrichment on outcomes such as satisfaction, performance have been well documented, little is known about the mechanisms underlying the relationships. Examining underlying and intervening mechanisms is necessary to understand comprehensively the relationships among work-family construct-related variables (Bansal and Agarwal, 2020). As discussed above, support of border keepers positively predicts work-family enrichment. However, whether work-family boundary flexibility plays a mediating role between border keeper’s support and work-family enrichment is still under discovered.

Lastly, it has also been noted that most of the work-family studies were conducted in a western context (Eby et al., 2005). However, while considering work-family interface, one needs to understand and appreciate that it is a culture-specific phenomenon (Powell et al., 2009). Different cultures often have different beliefs, values, and expectations (Albert and Ah Ha, 2004). Differences of individuals’ affective experiences in the workplace may be explained by the importance of family afforded in a cultural value (Del Campo et al., 2013) and organizational culture effect on work-family enrichment (Gordon et al., 2007). Therefore, generalizations to other contexts, which are totally different, for example China, are certainly questionable. Cultural belief especially gender culture in China suggests that the wife often contribute to provide non-monetary aid such as domestic work to the husband which is called “men outside and women inside.” So Chinese preschool teachers who are mostly female will face special situations and questions while they deal with work-family border. Therefore, it is important to examine work-family interface of preschool teachers in the unique context of China. Further, in the studies on the work-family boundary of preschool teachers in China, most of them focus on the work-family conflict and its consequences, and seldom study the work-family enrichment (Ji and Yue, 2020; Zhou et al., 2020). Women account for a large proportion of preschool teachers in China. Take China’s education data in 2019 as an example, there are 2,763,104 full-time teachers in preschool education institutions, of which 2,702,111 are female full-time teachers, accounting for 97.97%. Moreover, the expectation of women in Chinese traditional society is endowed with more family responsibilities such as raising children, supporting the elderly and “free nannies.” However, in the women’s liberation movement in modern China, “women can hold up half the sky,” and contemporary Chinese women are not only responsible for many and heavy family roles, but also given the responsibility of supporting their families and taking one side alone. This study takes preschool teachers in China as a sample and extends the study of work-family enrichment to Chinese context, especially focusing on the influence of border keeper’s support (work support and family support) on work-family enrichment for preschool teachers in China.

**FIGURE 1:**
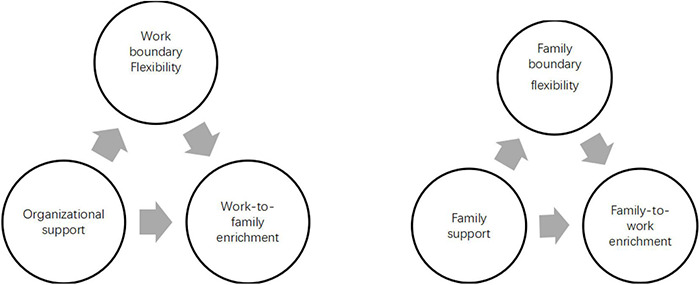
Research hypothesis.

The current research drew on the work-family border perspective to expand existing theoretical understanding of the relationship of the border keeper’s support and work-family enrichment. In particular, it examined the process by which support affects enrichment through a chain mediation model involving work-family boundary elasticity. Therefore, by testing the hypothesized theoretical model (Figure 1), this research provided a more comprehensive examination of the underlying relationships linking border keeper’s support to work-family boundary elasticity, and finally, work-family enrichment.

## Theoretical Framework and Literature Review

According to work-family border theory, different roles that individuals acting in work and family domains need role transitions as a boundary-crossing activity, where one exits and enters roles by surmounting boundaries based on border theory (Ashforth et al., 2000). Although participating in multiple roles will lead to resource drain (Greenhaus and Beutell, 1985), it will bring positive outcomes to employees, organizations and families (Greenhaus and Powell, 2006; McNall et al., 2010).

Since enrichment often refers to positive spillover, it is necessary to analyze the concept of spillover. Spillover conceptually represents the process whereby behaviors, moods, stress, and emotions from one realm of social life affect those in another and vice versa (Staines, 1980; Lawson et al., 2013). The spillovers from family to work environment can be divided into educational spillover and psychological spillover (Crouter, 1984). Educational spillover occurs when the individual learns something at home, a skill, an attitude, or a perspective, that can be applied elsewhere, including on the job. Psychological spillover, on the other hand, is a more transitory phenomenon. It includes the ways in which family life affects an individual’s energy level, attention span, and mood that, in turn, are brought into the work setting by the worker (Crouter, 1984). The spillover relationship is based on the similarity (or congruence) in their perceptions of peoples’ work and non-work experiences.

Positive spillover refers to the process by which what happens in one domain often spills back over to the other domain. These processes imply a transference of the acquisition of gains in one domain (e.g., work) to the use of these gains in the other domain (e.g., home; Grzywacz and Marks, 2000). Work-family positive spillover have been conceptualized as: (1) developmental gains, referring to skills, knowledge, values, or perspectives; (2) affective gains, referring to aspects of emotion; (3) capital gains, referring to economic, social, or health assets; and (4) efficiency gains, referring to the enhanced focus or attention induced by multiple role responsibilities (Crouter, 1984; Hobfoll, 2001).

According to work-home resource theory (W-HR) (ten Brummelhuis and Bakker, 2012), enrichment is described as a process of resource accumulation: work and family resources increase personal resources, which in turn can be used to improve family and work outcomes (Heras et al., 2021). W-HR model also explains how conditional factors such as personality, personal coping strategies, culture, general wealth conditions, public policies, trade unions, cultural norms on participation in work and social equality affect the occurrence of work-family conflicts and enrichment. The theory allows us to examine how work-family conflicts and enrichment develop (ten Brummelhuis and Bakker, 2012). Wayne et al. (2007) offered two broad categories of resources related to the enrichment process: personal characteristics and environmental factors. Research to date has focused on environmental influence related to work-family enrichment, such as supervisor support (Wattoo et al., 2018), co-worker support and family support (Nasurdin and O’Driscoll, 2012). Social support at work from supervisors and coworkers is a resource that can enhance performance and well-being in the family (Frone et al., 1997). Research has consistently demonstrated the positive consequences of social support such as reduced perceptions of role stressors and time demands at work and increased satisfaction and well-being (Parasuraman et al., 1992; Carlson and Perrewé, 1999). Having fewer organizational time demands is an aspect of culture related to more enrichment from work to family (Wayne et al., 2006). Thus, having supportive coworkers, employers, and/or a family-friendly work environment may lead to more positive affect, a sense of energy (Marks, 1977), or confidence from work which carries over and enhances functioning of the family. Hobfoll (2001) found personal characteristics are those traits or skills that result from one’s orientation to the world such as self-esteem and optimism. Self-efficacy and other factors bring within-domain and cross-domain spillover effects of work-family enrichment on job and family satisfaction (Westman et al., 2009). WFE and FWE were positively related to self-efficacy.

Work-family enrichment provides employees with sufficient resources to meet their work and family needs, so that they feel motivated to participate in various activities in the workplace and tend to show more creative work behaviors (Bansal and Agarwal, 2020). A meta-analysis shows that although both WFE and FWE are related to job satisfaction, organizational commitment, etc.; work-to-family facilitation is more related to job-related variables, and family-to-work facilitation is more related to non-job-related variables. In addition, the relationship between work and family seems to depend on moderating variables, including the proportion of women in the sample and structural tags (e.g., gain, promotion, positive spillover) (McNall et al., 2010).

According to previous studies, the antecedents of work-family enrichment include: role input (including time, energy and emotion input), resource accumulation, functional improvement (improvement of means and skills to solve corresponding problems), family-friendly organizational culture (career support, supervisor support and flexible working hours), job characteristics such as job decision-making autonomy and flexible working hours (Greenhaus and Powell, 2006; ten Brummelhuis and Bakker, 2012; Mauno and Rantanen, 2013; Bansal and Agarwal, 2020). Others emphasized that personal characteristics such as self-confidence or self-evaluation (Boyar and Mosley, 2007) and positive emotion was positively correlated with work-family enrichment (Tement and Korunka, 2013). According to Bhargava and Baral (2009), core self-evaluations (personality), family support, supervisor support and job characteristics are the antecedents of work-family enrichment, and work-family enrichment is positively correlated with work or family satisfaction, organizational commitment, and organizational behavior. According to French et al. (2018), work support and family support are key variables of work-family enrichment. It is proposed that enrichment occurs through two pathways: the instrumental pathway occurs when the resources gained in one role directly promote higher performance in the other role, and the affective pathway occurs when resources acquired from one role generate positive emotions, which indirectly facilitate functioning and performance in the other role (Carlson et al., 2006). Zhou et al. (2020) pointed out that in the workplace, supervisors and colleagues can provide specific instrumental support, while family’s support is usually emotional support (such as encouragement and confirmation).

### Relationship Between Organizational Support and Work-To-Family Enrichment

Organizational support, both formal policies and informal organizational and supervisory support including family-supportive organizational culture has been suggested to not only help employees balance work and family domains, but also has been shown to benefit the organization (Del Campo et al., 2013) and the family. As mentioned earlier, border keeper’s support as an important resource (e.g., from spouses or supervisors), contribute to perceptions of enrichment (McNall et al., 2011; Michel and Clark, 2013; Nicklin and McNall, 2013) with personal resources’ accumulation, directly or indirectly through border flexibility. For employees, work support (also called organizational support) may be the most important source of social support in general (French et al., 2018). Organizational support is negatively correlated with work-family conflict and turnover intention (Ford et al., 2007; Zhou et al., 2020) and helps employees achieve work-family enrichment and organizational commitment of employees (Martin et al., 2002). Chen and Powell (2012) also confirmed that in Chinese society, there is a positive correlation between the acquisition of work role resources and work-family enrichment (Chen and Powell, 2012).

Previous studies have mainly explored the effects of family-friendly/supportive organizational culture, job autonomy, flexible working hours, supervisor support, career support and other organizational support factors on work-family enrichment. There is a positive correlation between family supportive organizational culture or work atmosphere and work-family enrichment (Bansal and Agarwal, 2020). Family-supported organizational culture can provide individuals with a more flexible working environment and meet the individual family needs, which has a good impact on individual emotions and realizes work-family enrichment (Wayne et al., 2006). Family-friendly organizational culture may provide resources, making employees feel valued, reducing employees’ stress and willingness to leave, thus accumulating personal resources and realizing mutual facilitation between work and family fields (Heras et al., 2021).

In addition, work autonomy and flexible working hours are positively correlated with work-family enrichment (Bhargava and Baral, 2009; Odle-Dusseau et al., 2012; Bansal and Agarwal, 2020). Having job autonomy and working with a supportive boss can enhance employees’ perceptions of balance between demands faced in the work and family domains and resources at their disposal (ten Brummelhuis and Bakker, 2012).

Finally, previous research shows that supervisor support is positively related to work-family enrichment (Bhargava and Baral, 2009; Odle-Dusseau et al., 2012^;^ Bansal and Agarwal, 2020). In recent years, POS (perceived organizational support) has been proposed to distinguish between objective existence and subjective perception of organizational support, and POS is the result of objective working conditions (such as training) (Heras et al., 2021). Zhang et al. (2012) pointed out that the perceived service-oriented leadership level of individuals is significantly related to work-family enrichment, and the work environment that allows sharing and communication of family problems is also closely related to the generation of work-family enrichment (Zhang et al., 2012). Perceived organizational support creates resources for employees, promoting employees’ organizational identity and sense of value (Nicklin and McNall, 2013; Heras et al., 2021).

### Relationship Between Family Support and Family-To-Work Enrichment

Family support is the antecedent of work-family enrichment (Bhargava and Baral, 2009). According to McNall et al. (2010), there is a strong correlation between FWE and family support. Family support may make employees have positive emotional state and attitude toward family and work at the same time, and help employees keep the balance between work and family (Cordes and Dougherty, 1993), thereby increasing their job satisfaction (Kwok et al., 2015) and organizational commitment (Zhang et al., 2015). Sonnentag et al. (2010) pointed out that positive family members’ interaction and good emotional communication can have positive effects on individual work. At the same time, family support can reduce work-family conflict by preventing family from interfering with work excessively (Zhou et al., 2020).

As a female-dominated profession, preschool teachers are required to play multiple roles as teachers, wives, mothers and daughters at the same time. Especially in China, preschool teachers’ social status is relatively low, and they don’t feel much support from their families. On the contrary, their families don’t understand their work, which leads to work-family conflicts. This work-family conflict has been well documented as a major stressor for teachers and increases their willingness to leave (Cinamon et al., 2007). Supportive family can help preschool teachers deal with work-family conflicts, improving their organizational commitment, and thus reducing their turnover intention (Zhou et al., 2020).

### Influence of Boundary Flexibility on Work-Family Enrichment

An important variable in the study of work-family border is boundary flexibility (Bulger et al., 2007). The higher level of job boundary flexibility, the smaller the perceived work-family conflict, and vice versa (Clark, 2000). With the accumulation or cross-border use of personal resources, job flexibility well explains how organizational support solves the contradiction between work-family needs and realizes work-family enrichment (Voydanoff, 2004). Flexible working hours are a resource provided to employees (Chen and Fulmer, 2018), which can reduce employees’ cognitive failures in work and family by increasing their perceptual control in these two fields (Hsu et al., 2021) and are a stronger predictor of work-family enrichment (Rastogi et al., 2016).

Supporting family can make the boundaries between home and work domains flexible. If family members are willing to take on more responsibilities in the family area (such as sharing housework or picking up children), employees can save more time and energy from the family field to deal with their own work responsibilities, thus reducing the interference of family to work and reducing the risk of work-family conflict (Clark, 2000). Family support shows understanding of employees’ work and provides emotional support, which can reduce employees’ guilt toward their families (Martin et al., 2002).

Boundary theory argued that in the process of role transformation between work and family, individuals must negotiate with important members in the two domains on the boundaries between roles and domains. In the negotiation process, those members who have influence on determining the border are the border keepers (Clark, 2000). In general, supervisors are the most prevalent keepers of borders in the workplace, while spouses are the main border-keepers in the family. If consensus is reached, individuals may gain more social support to promote work-family enrichment.

Based on the existing research, this study puts forward the following hypothesis:

Hypothesis 1: Organizational support directly affects WFE.
Hypothesis 2: Work boundary flexibility mediate the relationships between organizational support and WFE.
Hypothesis 3: Family support directly affects FWE.
Hypothesis 4: Family boundary flexibility mediates the relationships between family support and FWE.

The research assumptions are shown in Figure 1.

## Methods

### Participants

In this study, preschool teachers in Guangdong Province of China were selected as the research participants, and 674 questionnaires were collected. In total, 504 valid questionnaires were obtained, and the effective recovery rate was 74.77%. The questionnaire includes demographic variables, work-family enrichment, border keepers’ support and boundary flexibility. In the sample, there are 14 male preschool teachers (2.78%) and 490 female preschool teachers (97.22%). And the sample consisted of 228 (45.24%) preschool teachers are living in rural areas. The specific distribution of other demographic variables of the participants, such as age, marital status is shown in Table 1.

**TABLE 1:** Demographics of participants (*N* = 504).

Demographic characteristic	Code in SPSS	*N*	%
**Gender**	0		
Male	1	14	2.78
Female		490	97.22
**Age**			
<25	1	135	26.79
26–30	2	123	24.4
31–35	3	97	19.25
36–40	4	69	13.69
41–45	5	54	10.71
**Professional title**			
No title	1	322	63.89
Third title	2	43	8.53
Second title	3	81	16.07
First title	4	49	9.72
Senior title	5	9	1.79
Position			
Assistant teacher	1	207	41.07
Head teacher	2	211	41.87
Grade/teaching and research group leader	3	22	4.37
Administrative positions such as deputy director	4	42	8.33
Other position	5	22	4.37
**Marital and fertility status**			
Unmarried (*n* = 204)	1	149	29.56
Married without children (*n* = 32)	2	23	4.56
Married with children (*n* = 343)	3	319	63.29
Divorced without children (*n* = 2)	4	1	0.2
Divorced with children (*n* = 14)	5	12	2.38
*bianzhi* (budgeted post)			
Owned	1	91	18.06
Non-owned	2	413	81.94
**Residence of preschool**			
City or township	1	276	54.76
Rural	2	228	45.24
**Types of preschool**			
Public (*n* = 230)	1	196	38.89
Private (*n* = 242)	2	203	40.28
Private inclusiveness (*n* = 123)	3	105	20.83

### Measures

#### Demographic Questionnaire

The demographic questionnaire was to collect the teachers’ basic information, including teachers’ age, gender, professional title, position, marital and fertility status, *bianzhi* (budgeted post), residence of kindergartens, and types of kindergartens. As unique features of teacher management system in China, *bianzhi* means legitimate access to governmental subsidies and benefits. Considering the possible link between demographic variables and teachers’ work-family enrichment, all the demographic variables were included as covariates.

#### Work-Family Enrichment Scale

The work-family enrichment scale developed by Grzywacz and Marks (2000) is used in this study. The scale consists of six items in two dimensions: WFE and FWE, such as “communication with family helps me solve problems at work.” Rickett’s score of 5 points is used, with 1 indicating “very inconsistent” and 5 indicating “very consistent.” The higher the score, the higher the work-family enrichment level. In this study, confirmatory factor analysis showed that the single-factor model fitted the data well, with χ^2^ (8, *N* = 504) = 40.214, CFI = 0.969, TLI = 0.941, SRMR = 0.043, RMSEA = 0.089, and the 90% confidence interval of RMSEA was [0.063, 0.118]. In this study, the Cronbach’s α coefficient of the questionnaire is 0.796.

#### Boundary Flexibility Scale

This study adopts the boundary flexibility scale (Matthews and Barnes-Farrell, 2010), which consists of 12 items in four dimensions of two sub-scales: Work boundary flexibility (including ability of work elasticity and willingness of work elasticity), and Family boundary flexibility (including ability of family elasticity and willingness of family elasticity), such as “If I need to deal with family or personal affairs, I can leave work early.” Likert 5 points are used to score, 1 means “very inconsistent” and 5 means “very consistent.” The higher the score, the higher the boundary flexibility level. In this study, confirmatory factor analysis showed that the single-factor model fitted the data well, with χ^2^ (98, *N* = 504) = 250.580, CFI = 0.962, TLI = 0.954, SRMR = 0.047, RMSEA = 0.056, and the 90% confidence interval of RMSEA was [0.047, 0.064]. In this study, the Cronbach’s α coefficient of the questionnaire is 0.790.

#### Border Keepers’ Support Scale

According to the work-family border theory, border keeper’s support includes organizational support and family support. In this study, Rickett scored 5 points, 1 means “very inconsistent” and 5 means “very consistent.” The higher the score, the higher the support level of border keepers. The organizational support scale in this study is compiled based on five items in the perceived organizational support scale (POSS) developed by Eisenberger (Rhoades and Eisenberger, 2002) and the employ support for family scale (Friedman and Greenhaus, 2000), such as “My kindergarten cares about teachers’ thoughts.” In this study, a question was deleted during the revision of model fitting. Confirmatory factor analysis showed that the single-factor model fitted the data well, with χ^2^ (5, *N* = 595) = 5.588, GFI = 0.997, TLI = 0.99, SRMR = 0.016 and RMSEA = 0.015. In this study, the Cronbach’s α coefficient of the questionnaire is 0.789. The family support scale of this study adopts six items selected from Lee Siew Kim and Seow Ling’s (2001) research scale on family support of working women in Singapore, such as “my family cares about my work results and affirms my efforts at work.” In this study, a question was deleted during the revision of the model fitting. CFA showed that the single-factor model fitted the data well, with χ^2^ (5, *N* = 504) = 124.273, CFI = 0.912, SRMR = 0.041, and the 90% confidence interval of RMSEA was [0.185, 0.251]. In this study, the Cronbach’s α coefficient of the questionnaire is 0.875.

### Procedure

Data were collected by a digital anonymous parent-reported questionnaire through an online crowdsourcing platform^1^ in Guangdong province, China. The questionnaire link was distributed via the social media APP WeChat (i.e., a prevailing interpersonal message communication application in China) primarily by sending survey postings in the preschools’ communication WeChat Groups and by sharing the survey postings on the personal WeChat Moments of preschool principals and teachers so that potential participants could see the positing.

It took about 6–10 min to fill out the entire internet-based survey. The anonymous e-questionnaires were filled on smart mobile phone or computers. And a questionnaire can be successfully submitted only when the entire questionnaire was filled out without missing any item; responses from each unique assigned ID on an electronic device could not be repeatedly submitted; and pausing and resuming the survey at any time were allowed. As soon as the questionnaire was submitted, preschool teachers would receive a reward of Chinese ¥ 2 (approximately US $ 0.3). Ultimately, 674 questionnaires were collected. We removed 170 questionnaires that took less than 350 s to complete and in which teachers gave invalid information. All procedures were approved by the authors’ home institution ethics review committee.

### Analytic Strategies

SPSS 25.0 and Mplus 8.0 were used to analyze the data. The data analysis steps are as follows: Firstly, SPSS 25.0 is used to analyze the descriptive statistics and correlation between variables. Secondly, on the basis of Mplus 8.0, the intermediary analysis based on structural equation is used to test the intermediary effect of boundary flexibility, which combines the advantages of sequential test method and Bootstrap method. The test step is divided into two steps: the first step is to construct the structural equation model from the independent variable of border keepers’ support to the dependent variable of work-family enrichment, and the second step is to construct the structural equation model after the intermediary variable of boundary flexibility. In this way, the results of sequential tests and the confidence interval of Bootstrap method can be calculated. Because the sample distribution of ab of mediating effect does not obey the normal distribution in general, the confidence interval of mediating effect is estimated by the non-parametric percentile Bootstrap method with deviation correction. In this study, a total of 5000 samples were constructed, and 95% confidence interval was obtained by calculation. If the confidence interval does not contain 0, it means that the result is statistically significant (Erceg-Hurn and Mirosevich, 2008). The fitting index of the model was composed of χ^2^, CFI, TFI, SRMR and RMSEA. When CFI and TLI were greater than 0.90 and SRMR and RMSEA were less than 0.08, the fitting of the model was good (Hu and Bentler, 1999).

## Results

### Common Method Deviation

In this study, SPSS 25.0 and Mplus 8.0 were used for statistical analysis and mediating effect test. Firstly, in order to avoid the common method bias, the questionnaire is controlled, the electronic questionnaire is filled anonymously, and some questions are set with reverse questions, etc. In order to further improve the rigor of the research, two methods are used to test the common method bias: (1) With Harman single factor test (Podsakoff et al., 2003), exploratory factor analysis of all variables without rotation shows that there are nine factors whose eigenvalues are greater than 1, and the variance interpretation percentage of the first common factor is 22.09%, which is far less than the critical value of 40%. (2) A more accurate single common method factor control method was used to test the common method bias, and all the measured items were loaded with a common potential factor. The results showed that the model fitted poorly: χ^2^ (527, *N* = 504) = 6266.681, CFI = 0.332, TLI = 0.289, SRMR = 0.141, RMSEA = 0.147 (Hu and Bentler, 1999). Therefore, the results of both methods show that there is no obvious common method bias in this study.

### Description Statistics and Correlation Matrix

The mean (M), standard deviation (SD) of each variable and the correlation coefficient between variables are shown in Table 2. The results show that the average score of organizational support is 3.49 (*SD* = 0.505), and the average score of family support is 3.92 (*SD* = 0.707), which is higher than the median value of 3, indicating that the organizational support and family support of preschool teachers are at a high level, and the strength of family support is higher than organizational support; The average score of WFE was 3.21 (*SD* = 0.858), and the average score of FWE was 3.851 (*SD* = 0.767), which was higher than the median value of 3, indicating that the work-family enrichment of preschool teachers was at a high level, and the level of FWE is higher than that of WFE. The average score of work boundary flexibility is 2.02 (*SD* = 0.73), which is lower than the median value of 3, and the average score of family boundary flexibility is 3.50 (*SD* = 0.787), which is higher than the median value of 3, indicating that preschool teachers’ work boundary flexibility is at a low level and family boundary flexibility is at a high level.

**TABLE 2:** Descriptive statistics and correlation matrix of main research variables (*N* = 504).

	M ± SD	1	2	3	4	5	6
1. Organizational support	3.49 ± 0.505	1					
2. Family support	3.923 ± 0.707	0.326***	1				
3. Work-to-family enrichment	3.211 ± 0.858	0.284***	0.310***	1			
4. Family-to-work enrichment	3.851 ± 0.767	0.319***	0.529***	0.416***	1		
5. Work boundary flexibility	2.024 ± 0.73	−0.108*	−0.093*	0.106*	–0.087	1	
6. Family boundary flexibility	3.502 ± 0.787	0.308***	0.279***	0.247***	0.368***	0.001	1

*p < 0.05, **p < 0.01, ***p < 0.001.

Organizational support was positively correlated with WFE (*r* = 0.284, *p* < 0.001), and negatively correlated with work boundary flexibility (*r* = −0.108, *p* < 0.05); WFE was positively correlated with work boundary flexibility (*r* = 0.106, *p* < 0.05); Family support was positively correlated with FWE and family boundary flexibility (*r* = 0.529, *p* < 0.001; *r* = 0.279, *p* < 0.001); There is a positive correlation between FWE and family boundary flexibility (*r* = 0.368, *p* < 0.001), as shown in Table 2.

### Variance Analysis

The mean difference test was conducted for the two dimensions of work-family enrichment (WFE and FWE). In terms of WFE, there were significant differences for age, *F* = 3.039, *p* < 0.05. *Post hoc* analysis using the Scheffé *post hoc* criterion for significance indicated that the WFE of preschool teachers aged 26–30 years old (*M* = 3.34, *SD* = 0.87) was significantly higher than who under 25 years old (*M* = 3.01, *SD* = 0.77). There were significant differences for marital and fertility status, *F* = 2.611, *p* < 0.05. *Post hoc* analysis showed that the score of married preschool teachers with children (*M* = 3.30, *SD* = 0.90) was significantly higher than that of unmarried preschool teachers (*M* = 3.07, *SD* = 0.75). The WFE was significant in the residence of preschool, *t* = 5.042, *p* < 0.05, score of preschool teachers from rural areas (*M* = 3.29, *SD* = 0.85) was significantly higher than that of preschool teachers from urban areas (*M* = 3.12, *SD* = 0.86). In terms of FWE, there was significant difference for age, *F* = 3.778, *p* < 0.01. *Post hoc* analysis showed that the score of preschool teachers under 25 years old (*M* = 3.63, *SD* = 0.79) was lowest. There was significant difference in marital and fertility status, *F* = 3.506, *p* < 0.01. *Post hoc* analysis indicated that score of married preschool teachers with children (*M* = 3.93, *SD* = 0.75) was significantly higher than that of unmarried preschool teachers (*M* = 3.66, *SD* = 0.78).

### Mediation Test of Boundary Flexibility

#### Mediation Test of Family Boundary Flexibility

**FIGURE 2:**
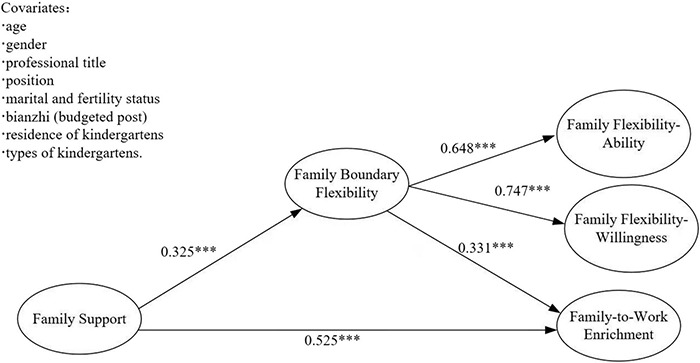
Influence of family support on FWE: family boundary flexibility as the intermediary.

The confidence interval of each coefficient was estimated by the non-parametric percentile Bootstrap method with deviation correction. In the first step, the direct effect of family support (FS) on FWE was examined. The results showed a good fit, χ^2^ (19, *N* = 504) = 158.909, CFI = 0.934, TLI = 0.908, SRMR = 0.033, RMSEA = [0.088, 0.118]. After controlling the covariates such as age of preschool teachers, the direct predictive effect of family support on FWE was significant (β = 0.645, *p* < 0.001), with a 95% confidence interval [0.561, 0.703]. In the second step, the family boundary flexibility (FBF) was added to the original model. The results showed that the model also fitted well, χ^2^ (114, *N* = 595) = 354.204, CFI = 0.947, TLI = 0.937, SRMR = 0.050, RMSEA = 0.065. As shown in Figure 2, after controlling the age of kindergarten’s teachers, the predictive effect of family support on family boundary flexibility was significant (β = 0.325, *p* < 0.001), with 95% confidence interval [0.195, 0.453]. The predictive effect of family boundary flexibility on FWE was also significant (β = 0.331, *p* < 0.001), with 95% confidence interval [0.205, 0.461]. The predictive effect of family support on FWE was still significant (β = 0.525, *p* < 0.001), with 95% confidence interval [0.411, 0.631]. The mediating effect quantity was 0.108, *p* < 0.001, the 95% confidence interval was [0.052, 0.173], the mediating effect ratio (ab/c) was 17.06%, the direct effect ratio was 82.94%. Therefore, the family boundary flexibility has a significant mediating effect between family support and FWE, and the hypothesis holds. See Table 3 for details.

**TABLE 3:** Direct and indirect effects of family and organizational support on work-family enrichment.

Effect	β	*SE*	*p*	95% CI
Family boundary flexibility as mediator				
Direct effects				
Family support→family-to-work enrichment	0.525***	0.056	0.000	[0.411, 0.631]
Indirect effects				
Family support→family boundary flexibility→family-to-work enrichment	0.108**	0.031	0.001	[0.052,0.173]
Work boundary flexibility as mediator				
Direct effects				
Organizational support→work-to-family enrichment	0.346***	0.062	0.000	[0.221, 0.462]
Indirect effects				
Organizational support→work boundary flexibility→work-to-family enrichment	−0.03	0.017	0.074	[−0.066, 0.000]

*p < 0.05, **p < 0.01, ***p < 0.001.

*SE, standard error; 95% CI, 95% confidence interval.*

#### Mediation Test of Working Boundary Flexibility

**FIGURE 3:**
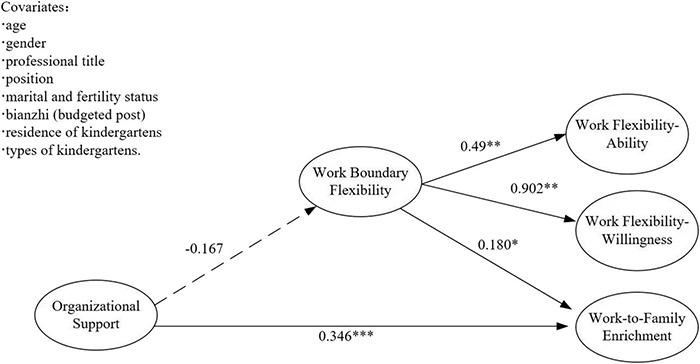
Influence of OS on WFE: work boundary flexibility as the intermediary.

In the first step, the direct effect of organizational support on WFE was examined. The results showed a good fit with χ^2^ (40, *N* = 504) = 53.389, CFI = 0.991, TLI = 0.988, SRMR = 0.033, RMSEA = 0.026. After controlling for demographic variables such as the age of preschool teachers, the direct predictive effect of organizational support on WFE was significant (β = 0.314, *p* < 0.001), with a 95% confidence interval [0.213, 0.407]. In the second step, the working boundary flexibility was added to the original model. The results showed that the model also fitted well with χ^2^ (114, *N* = 504) = 235.555, CFI = 0.962, TLI = 0.955, SRMR = 0.054, RMSEA = 0.046. As shown in Figure 3, after controlling the covariates such as age of preschool teachers, the predictive effect of organizational support (OS) on work boundary flexibility was not significant (β = −0.167, *p* > 0.05), while the predictive effect of work boundary flexibility on WFE was significant (β = 0.180, *p* < 0.05). The 95% confidence interval was [0.041, 0.304]. The predictive effect of organizational support on WFE was significant (β = 0.346, *p* < 0.001), and the 95% confidence interval was [0.221, 0.462]. The mediating effect dose was −0.030, *p* > 0.05, the 95% confidence interval was [−0.066, 0.000]. Therefore, the mediating effect of work boundary flexibility between organizational support and WFE is not significant, and the hypothesis is not valid, as shown in Table 3.

## Discussion

The present study investigates the current situation of work-family enrichment in Chinese preschool teachers and the relationships among border keepers’ support, boundary flexibility and work-family enrichment. As expected, organizational support was positively correlated with WFE directly; family support was positively correlated with FWE; the family boundary flexibility has a significant mediating effect between family support and FWE; the mediating effect of work boundary flexibility between organizational support and WFE is not significant. The results mostly confirm our hypotheses.

### Work-Family Enrichment, Organizational Support, Family Support and Boundary Flexibility of Preschool Teachers

This study found that the work-family enrichment of preschool teachers is on the upper-middle level, and the score of FWE is higher than WFE, which is consistent with the existing research results. Work boundary flexibility is moderately low, while family boundary flexibility is moderately high. There are not only cultural reasons, but also the characteristics of preschool teachers’ profession. There is a blurring of the boundary between work and family roles in Chinese workplaces due to the influence of traditional culture (Chan, 1996; Luo et al., 2012; Liu et al., 2015). The Chinese culture is typically collectivistic cultures which means employees tend to comply with authority, and therefore are inclined to do what their bosses say, and are generally more willing to accept high work demands (Luo et al., 2012). Under this cultural background, it is easy to blur the boundary between family and work because of the continuity of preschool teachers’ work in time and space. It leads to a certain degree of asymmetric penetration in the boundary between work and family. Compared with the strict institutional norms of the organization, the flexibility of family boundaries is greater, so preschool teachers often occupy resources in the family field, such as taking home the work that cannot be completed during working hours, which increases the flexibility of family boundaries and gains more from family-to-work. This study also found that organizational support is at an upper-middle level, which is consistent with existing studies. Family support is in the upper middle level, which is different from the previous research which show that the family support level of preschool teachers in China is low (Cinamon et al., 2007). The results of this study may be related to the professionalization of preschool teachers in China these years (Zhang et al., 2020). The significance and value of preschool education have been paid attention to, the professional value of preschool teachers has been widely recognized and affirmed, and the social reputation of preschool teachers is constantly improving. And the implementation of “National Training Plan” and “Provincial Training Plan” has improved the professional development level of preschool teachers, enhanced their professional self-esteem and professional self-confidence. Therefore, the level of organizational support and family support is at a high level.

### The Relationship Among Work-Family Enrichment, Organizational/Family Support and Work/Family Boundary Flexibility of Preschool Teachers

It is found that there is a significant direct influence between family support and FWE of preschool teachers, and the intermediary role of family boundary flexibility between family support and FWE exists. There is a significant direct influence between organizational support and WFE of preschool teachers, but the mediating effect of work boundary flexibility between organizational support and WFE does not exist.

#### The Influence of Family Support and Family Boundary Flexibility on Family-to-Work Enrichment

First of all, family support has a significant positive predictive effect on FWE. Previous studies have also confirmed that family support, such as sharing housework or picking up children, or showing understanding and providing emotional support, can reduce individual guilt toward the family. At the same time, it will make individuals have positive feelings about family life and work, which will help them better perform their duties and promote their work development (Cordes and Dougherty, 1993). Similarly, according to the work-family border theory, family support can make individuals maintain a positive emotional state in the family field and form an optimistic attitude toward life, which can penetrate into the work field and help them look at work positively, thus improving the level of FWE (Zhang et al., 2015; Lambert et al., 2016). In China’s collectivist society, influenced by the traditional Chinese social concept of “putting career first,” families have always been expected to provide strong support for work. Studies have shown that family support seems to play an important role in motivating employees to work hard (Tang et al., 2014). Therefore, the level of FWE will increase with the increase of family support.

Secondly, family support has a significant positive predictive effect on family boundary flexibility. According to the work-family border theory, family support can make the boundary between family domain and work domain flexible (Clark, 2000). In other words, family support makes individuals feel that their role transformation or domain crossing at any time is accepted, understood and tolerated. If individuals receive this signal, even if there are other factors in the family that affect border crossing (there are old, weak, women and children to take care of, and there is less time at home), the resistance of border crossing can be weakened by individuals. For example, preschool teachers perceive their spouses’ recognition of their profession and their attitude of full support, so teachers can still switch to the work field at any time when facing the conflict between caring for children at home and work, that is, family support can promote the improvement of FWE level.

Finally, the family boundary flexibility has a significant positive predictive effect on the FWE, which is consistent with the previous research results (Clark, 2000). According to the work-family border theory, the boundary between work and family is flexible. If family members are willing to take on more responsibilities in the family field (such as sharing housework or picking up children), individuals can save more time and energy from the family field to deal with their own work responsibilities, thus improving the FWE. This is especially true in China. Chinese Traditional culture emphasizes material success versus quality of life. East Asian including Chinese workers place a strong emphasis on their careers in order to contribute to the success of their family. They are typically supported by their family to do so for the family’s Long-term benefits (Le et al., 2020).

#### The Influence of Organizational Support and Work Boundary Flexibility on Work-to-Family Enrichment

Firstly, organizational support has a significant positive predictive effect on WFE, which is consistent with previous research results (Bhargava and Baral, 2009; Wattoo et al., 2018). French et al. (2018) pointed out that organizational support may be the most important support resource. This kind of resource is beneficial for teachers to be more handy when facing and dealing with work or family problems, which will make individuals show more positive emotions and behaviors, and then promote the harmonious development of families and work. Similarly, Powell and Greenhaus (2006) believed that organizational support resources obtained at work (such as respect and care) may stimulate better job performance, thus having a more positive impact at work, and finally transforming into a more positive impact in the family field, thus enhancing WFE (Powell and Greenhaus, 2006).

Secondly, organizational support has no significant positive predictive effect on job boundary flexibility, different from previous research results. Previous studies believe that organizational support, as an important resource, can make the boundary between family domain and work domain more flexible (Nicklin and McNall, 2013). The reason may be related to the particularity and present situation of preschool teachers’ profession in China. Preschool teachers in China are recognized as occupations with heavy workload, relatively scarce and living in the strict organizational system, which leads to the low flexibility of the working boundary of preschool teachers (Zhang et al., 2020). The results of this study also confirm this view: the average value of working boundary flexibility is 2.024 points, which is in the middle and lower level. The organizational support provided by Chinese kindergartens is more emotional support, such as caring for teachers’ ideas and family life, recognizing teachers’ efforts, etc., and less instrumental support (Zhou et al., 2020). Therefore, in this study, the level of organizational support is high, but it can’t give preschool teachers actual flexible time and independent control in realistic conditions. That is to say, the organizational support that Chinese kindergartens can provide for preschool teachers is more family-friendly organizational culture and humanistic care. According to Blau’s (1964) theory of social exchange, in the employer-employee relationship, when one party feels favorable treatment, the other party will also give back, thus bringing favorable results to both parties (Rhoades and Eisenberger, 2002). Applying this to the work-family interface, employees are likely to feel supported and cared for by their organization when they feel the organization is helping them manage their work and family roles (Rhoades and Eisenberger, 2002; Aryee et al., 2005). Many studies in China have drawn on exchange-related theories such as organizational support theory, and social exchange theory to argue that if an organization provides family-friendly work policies and practices, employees will feel that the organization support them, and will reciprocate by being more committed to the organization and exerting greater effort. This may be because that loyalty to organizations and reciprocation of positive behavior is well aligned with work values that are emphasized in China society (Le et al., 2020). Preschool teachers in China who are deeply influenced by traditional culture believe that they should repay the support of organizations or supervisors by working hard based on reciprocity of social belief (Siu et al., 2015).

Thirdly, the elasticity of work boundary has a significant positive predictive effect on WFE. Similar to previous studies, job flexibility helps employees to better manage their family life, resulting in higher productivity and satisfaction, and then affecting individuals’ positive emotions, emotions and behaviors in the family field (Bailyn, 1993). According to Greenhaus and Powell (2006), it is believed that positive emotions generated by roles in work or family can indirectly improve performance in another role field (Greenhaus and Powell, 2006). Individuals with high positive affectivity are more likely to experience the two-way gain of work and family, and individuals inclined to self-integration are more likely to experience WFE (rather than FWE). Among the discussions on mechanisms underlying the relationships among work-family-related variables, extant literature has yielded mixed findings regarding gender and gender role attitude effect on the relationships (Byron, 2005). Additionally, gender role attitude affects an individual’s identity and behavior, the role she/he chooses to enact, and how she/he effectively chooses to enact them (Eagly and Wood, 2011). According to Eagly’s social role theory (1987), men and women have different preferences for work and family roles, which is the result of gender role socialization (Eagly, 1987). For example, women are more likely to integrate work and family roles, while men are more likely to separate or mentally separate these roles (Andrews and Bailyn, 1993). Using structural equation modeling, Rothbard (2001) found that women have more work-family connections than men, and women experience more enrichment from home to work. Preschool teachers are mainly women, and they generally tend to integrate the work-family border.

## Implications

### Theoretical Implications

**FIGURE 4:**
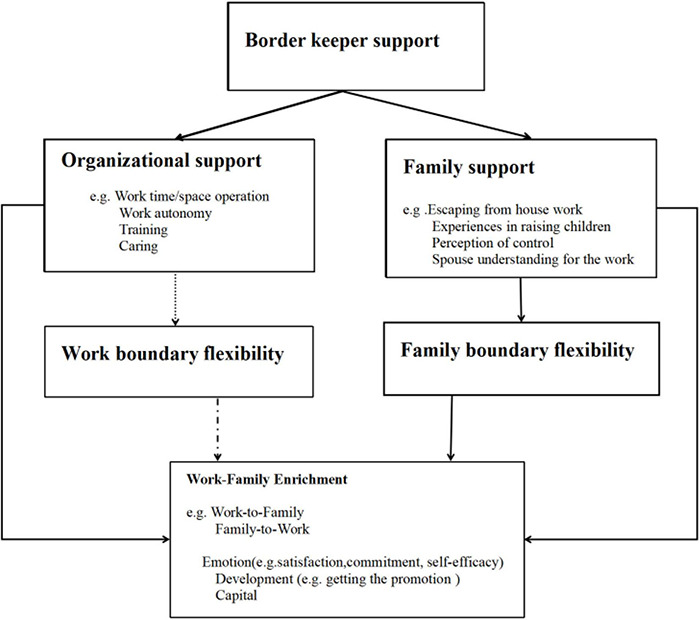
Theoretical mechanism between border keepers’ support and work-family enrichment.

Theoretically, this study is based on the W-HR theory focusing on the positive synergistic potential of the work-family interface. Due to personal resources accumulated from border keeper’s support, employees achieved more flexibility and ability to deal with demands from work and family domain. This study has several implications: according to the resource-demand matching theory, the needs from work, family and the cross-border involve time-based demands, strain-based demands (job demands, job insecurity and various family needs) and role blurring. Organizational support (work autonomy, support from supervisors and colleagues, sense of work accomplishment, respect, pride, etc.) and family support can increase the resources that individuals may obtain. If the resources that individuals obtain can match the needs from the two fields of work and family, individuals are more likely to obtain mutual benefits between work and family. The present study extends the range of antecedents and outcomes of WFE by examining its relationship with border keepers’ support and border flexibility. The process and mechanism is summarized as shown in Figure 4.

### Practical Implications

The importance of the beneficial effects of border keepers’ support and border flexibility on work- family enrichment is supported by this study. Kindergartens should incorporate the concept of work-family reciprocity into the human resource management process, and use these results to teach their employees to deal with the relationships between work and family.

First of all, the study points out that the heavy work leads to the fuzzy work family boundary for preschool teachers, especially urban preschool teachers. And the low elasticity of work boundary which shows that preschool teachers need to deal with the increasing professional requirements with the performance reform and professional movement in recent years. Preschool teachers, especially those in urban kindergartens, are required to undertake multiple roles such as instruction, observation, doing research, writing articles and so on at the same time, with a large workload. It is suggested to reduce pressure and burden, increasing their abilities or power to realize the mutual gain of the two domains. Since Chinese society is a reasonable society, it is emphasized to give consideration to emotion and rationality, taking precedence over rationality. Therefore, the organization should first give teachers more professional autonomy, respect, emotional care, and instrumental support to improve teachers’ ability to use resources across borders by giving more professional development opportunities and various demand-based training.

Further, researches to date discussed the moderating effect of gender on the relationship between border flexibility and work-family enrichment. And kindergartens have always been dominated by female teachers, and preschool teachers are often viewed as “female work” (Zhang et al., 2019; Zhou et al., 2020). It is generally believed that women have the bounden duty of mother. Women’s characteristics enable them to play the role of teachers appropriately. This is easy to cause people to establish a connection between the stereotyped impression of teachers’ role and teachers’ professional identity (Britzman, 1991). According to social constructivism, the concept of women is not innate, but social construction. As revealed by Maxine Hong Kingston (a contemporary Chinese American female writer) in her famous work *the woman warrior* (1976), Chinese women have long been suppressed by male chauvinism (Eng, 2006). It is more socially acceptable for women to choose occupations with more flexible boundaries (Wayne et al., 2007). And higher flexibility of work boundary can promote the integration of work and family, and the flow of resources from work to family (Andrews and Bailyn, 1993; Rothbard, 2001). It’s necessary for kindergartens to give their employees more work flexibility, and change the gender culture to establish preschool teachers’ self-efficacy and job identity.

Lastly, contemporary Chinese social development highlights the contradiction between tradition and modernity. This contradiction is embodied in the work-family boundary of preschool teachers. On the one hand, female’s participation is increasingly emphasized in social construction, and the concept of “women half the sky” has been deeply rooted in the hearts of the people. Under this background, work is more important than family and preschool teachers’ willingness for family boundary elasticity is strong. At the same time, inter-generational child rearing is popular influenced by kinship social culture (Fei, 2015), and preschool teachers have the ability of strong flexibility in family boundaries. To help teachers cope with such a contradictory dilemma arising from multiple remands from work and family, teachers’ ability of value clarification should be improved.

### Limitations and Future Research

This study has the following shortcomings: firstly, the samples are mainly from Guangdong Province which is relatively small in scope. Secondly, the data in the current study were collected with self-report and cross section. Although the measurements were widely used in previous studies and were proved to be reliable and valid in the current study, might still have led to a subjective bias and difficulties to track the study. Consequently, it is recommended for future studies to adopt multiple evaluation methods and data (e.g., field observation, tracking studies) with reliable measurements to evaluate border keeper’s support, work-family enrichment, border flexibility.

While women and men differ in their experiences of WFE (Boz et al., 2016), this study did not adopt a gendered perspective as sampling difficulties caused by the small proportion of male preschool teachers. Future research should expand the scope of sampling and enrich the sources of data, using tracking research, such as using diary method to record the continuous state of work-family enrichment of preschool teachers under the changes of the times. And personal factor such as personal preference and gender consciousness should be investigated more explicitly.

## Conclusion

This study puts forward and tests the influence of border keeper’s support (organizational support and family especially spouse support) on work-family enrichment (WFE and FWE) based on samples of 504 preschool teachers in China. Results showed that work-family enrichment of preschool teachers is on the upper-middle level, and the score of FWE is higher than WFE which is originated from the expectation of individual’s role in Chinese society that individuals should be career-oriented. This study confirms that family support has a direct and significant positive predictive effect on FWE, and organizational support has a direct and significant positive predictive effect on WFE. In addition, the study found that family boundary flexibility mediates the relationships between family support and FWE whereas work boundary flexibility did not mediate the relationships between organizational support and WFE. The above research results are partly consistent with the existing research, and partly inconsistent, which is related to the profound influence of traditional culture in Chinese society and the current situation of preschool teachers in China. Such findings have important implications for improving the work-family enrichment of preschool teachers.

## Data Availability Statement

The data are not publicly available due to privacy or ethical restrictions. The data that support the findings of this study are available on request from the corresponding author.

## Ethics Statement

The studies involving human participants were reviewed and approved by South China Normal University. Written informed consent for participation was not required for this study in accordance with the national legislation and the institutional requirements.

## Author Contributions

QP designed the research and drafted the manuscript. CL collected and extracted data for analysis. LZ provided important ideas and substantial feedback for the study and edited the manuscript. All authors have approved the final version of this article.

## Conflict of Interest

The authors declare that the research was conducted in the absence of any commercial or financial relationships that could be construed as a potential conflict of interest.

## Publisher’s Note

All claims expressed in this article are solely those of the authors and do not necessarily represent those of their affiliated organizations, or those of the publisher, the editors and the reviewers. Any product that may be evaluated in this article, or claim that may be made by its manufacturer, is not guaranteed or endorsed by the publisher.

## Footnotes

1
https://www.wjx.cn

## References

[B1] Albert R. D. Ah Ha I. (2004). Latino/Anglo-American differences in attributions to situations involving touch and silence. *Int. J. Intercult.Relat.* 28 253–280. 10.1016/j.ijintrel.2004.06.003

[B2] Andrews A. Bailyn L. (1993). “Segmentation and synergy: two models of linking work and family,” in *Men, Work, and Family*, ed Hood J. C. (Newbury Park, CA: Sage), 262–275.

[B3] Aryee S. Srinivas E. S. Tan H. H. (2005). Rhythms of life: Antecedents and outcomes of work-family balance in employed parents. *J. Appl. Psychol.* 90 132–146. 10.1037/0021-9010.90.1.132 15641894

[B4] Ashforth B. E. Kreiner G. E. Fugate M. (2000). All in a day’s work: Boundaries and micro role transitions. *Acad. Manage. Rev.* 25 472–491.

[B5] Bailyn L. (1993). *Breaking the Mold: women, Men, and Time in the New Corporate World.* New York: Simon and Schuster.

[B6] Bansal N. Agarwal U. A. (2020). Direct and indirect effects of work-family enrichment: role of gender role ideology. *Int. J. Product. Perform. Manag.* 69 873–894. 10.1108/ijppm-10-2018-0370

[B7] Bhargava S. Baral R. (2009). Antecedents and consequences of work-family enrichment among indian managers. *Psychol.Stud.* 54:213. 10.1007/s12646-009-0028-z

[B8] Blau P. M. (1964). *Exchange and Power in Social Life.* New York: Wiley.

[B9] Boyar S. L. Mosley D. C. (2007). The relationship between core self-evaluations and work and family satisfaction: The mediating role of work-family conflict and facilitation. *J. Vocat. Behav.* 71 265–281.

[B10] Boz M. Martínez-Corts I. Munduate L. (2016). Types of combined family-to-work conflict and enrichment and subjective health in Spain: a gender perspective. *Sex. Roles* 74 136–153. 10.1007/s11199-015-0461-5

[B11] Britzman D. (1991). *Practice Makes Practlce: a Critical Study of Learning to Teach.* New York: Suny Press.

[B12] Bulger C. A. Matthews R. A. Hoffman M. E. (2007). Work and personal life boundary management: Boundary strength, work/personal life balance, and the segmentation-integration continuum. *J. Occup. Health Psychol.* 12 365–375. 10.1037/1076-8998.12.4.365 17953495

[B13] Byron K. (2005). A meta-analytic review of work – family conflict and its antecedents. *J. Vocat. Behav.* 67 169–198. 10.1016/j.jvb.2004.08.009

[B14] Carlson D. S. Kacmar K. M. Wayne J. H. Grzywacz J. G. (2006). Measuring the positive side of the work–family interface: Development and validation of a work–family enrichment scale. *J. Vocat. Behav.* 68 131–164.

[B15] Carlson D. S. Perrewé P. L. (1999). The role of social support in the stressor–strain relationship: An examination of work-family conflict. *J. Manag.* 25 513–540. 10.1016/S0149-2063(99)00013-6

[B16] Carmona-Cobo I. Blanco-Donoso L. M. Garrosa E. (2021). Daily Beneficial Effects of Work-to-Family Facilitation on Employees’ Recovery and General Health: Is More Work Engagement Always Better? *Front. Psychol.* 12:661267. 10.3389/fpsyg.2021.661267 PMC8319573 34335371

[B17] Chan A. (1996). Confucianism and development in East Asia. *J. Contemp. Asia.* 26 28–45. 10.1080/00472339680000031

[B18] Chen Y. Fulmer I. S. (2018). Fine-tuning what we know about employees’ experience with flexible work arrangements and their job attitudes. *Hum. Resour. Manage.* 57 381–395. 10.1002/hrm.21849

[B19] Chen Z. Powell G. N. (2012). No pain, no gain? A resource-based model of work-to-family enrichment and conflict. *J. Vocat. Behav.* 81 89–98. 10.1016/j.jvb.2012.05.003

[B20] Cinamon R. G. Rich Y. Westman M. (2007). Teachers’ occupation-specific work-family conflict. *Career Dev. Q.* 55 249–261. 10.1002/j.2161-0045.2007.tb00081.x

[B21] Clark S. C. (2000). Work/family border theory: A new theory of work/family balance. *Hum. Relat.* 53 747–770. 10.1177/0018726700536001

[B22] Cordes C. L. Dougherty T. W. (1993). A review and an integration of research on job burnout. *Acad. Manage. Rev.* 18 621–656. 10.2307/258593

[B23] Crouter A. C. (1984). Spillover from Family to Work: The Neglected Side of the Work-FamilyInterface. *Hum.Relat.* 37 425–441. 10.1177/001872678403700601

[B24] Del Campo R. G. Cook A. Arthur M. M. (2013). Cultural differences in work-family policies and perceptions of organizational support. *Employ. Respons Rights J.* 25 23–39. 10.1007/s10672-011-9188-9

[B25] Eagly A. H. (1987). *Sex Differences in Social Behavior: a Social-role Interpretation.* Hillsdale: Erlbaum.

[B26] Eagly A. H. Wood W. (2011). Social role theory. *Handb. Theor. Soc. Psychol.* 2 458–476.

[B27] Eby L. T. Casper W. J. Lockwood A. Bordeaux C. Brinley A. (2005). Work and family research in IO/OB:content analysis and review of the literature (1980-2002). *J. Vocat. Behav.* 66 124–197. 10.1016/j.jvb.2003.11.003

[B28] Eng R. S. H. (2006). *A Study of Maxine Hong Kingston’s “The Woman Warrior” Using Archetypal Criticism.* Dominguez Hills: California State University.

[B29] Erceg-Hurn D. M. Mirosevich V. M. (2008). Modern Robust Statistical Methods An Easy Way to Maximize the Accuracy and Power of Your Research. *Am. Psychol.* 63 591–601. 10.1037/0003-066x.63.7.591 18855490

[B30] Fei X. T. (2015). *Rural China.* Beijing: People’s Publishing House.

[B31] Ford M. T. Heinen B. A. Langkamer K. L. (2007). Work and family satisfaction and conflict: A meta-analysis of cross-domain relations. *J. Appl. Psychol.* 92 57–80. 10.1037/0021-9010.92.1.57 17227151

[B32] French K. A. Dumani S. Allen T. D. Shockley K. M. (2018). A Meta-Analysis of Work-Family Conflict and Social Support. *Psychol. Bull.* 144 284–314. 10.1037/bul0000120 29239632 PMC5858956

[B33] Friedman S. D. Greenhaus J. H. (2000). *Work and Family – Allies or Enemies? What Happens When Business Professionals Confront Life Choices*. New York: Oxford University Press.

[B34] Frone M. R. (2003). “Work–family balance” in *Handbook of Occupational Health Psychology.* eds Quick J. C. Tetrick L. E. (Washington: American Psychological Association), 143–162.

[B35] Frone M. R. Yardley J. K. Markel K. S. (1997). Developing and testing an integrative model of the work-family interface. *J. Vocat. Behav.* 50 145–167. 10.1006/jvbe.1996.1577

[B36] Gordon J. R. Whelan-Berry K. S. Hamilton E. A. (2007). The relationship among work–family conflict and enhancement, organizational work–family culture, and work outcomes for older working women. *J. Occup. Health Psychol.* 12 350–364. 10.1037/1076-8998.12.4.350 17953494

[B37] Greenhaus J. H. Beutell N. J. (1985). Sources of Conflict Between Work And Family Roles. *Acad. Manage. Rev.* 10 76–88. 10.5465/amr.1985.4277352

[B38] Greenhaus J. H. Powell G. N. (2006). When work and family are allies: A theory of work-family enrichment. *Acad. Manage. Rev.* 31 72–92. 10.5465/amr.2006.19379625

[B39] Grzywacz J. G. Carlson D. S. Kacmar K. Wayne J. H. (2007). A multi- level perspective on the synergies between work and family. *J. Occup.Organ. Psychol.* 80 559–574. 10.1348/096317906X163081

[B40] Grzywacz J. G. Marks N. F. (2000). Reconceptualizing the work-family interface: An ecological perspective on the correlates of positive and negative spillover between work and family. *J. Occup. Health Psychol.* 5 111–126. 10658890 10.1037//1076-8998.5.1.111

[B41] Heras M. L. Rofcanin Y. Escribano P. I. Kim S. Mayer M. (2021). Family-supportive organisational culture, work–family balance satisfaction and government effectiveness: evidence from four countries. *Hum. Resour. Manag. J.* 3 454–475. 10.1111/1748-8583.12317

[B42] Hobfoll S. E. (2001). The influence of culture, community, and the nested-self in the stress process: advancing conservation of resources theory. *Appl. Psychol.* 50 337–421. 10.1111/1464-0597.00062

[B43] Hsu Y. S. Chen Y. P. Shaffer M. A. (2021). Reducing Work and Home Cognitive Failures: the Roles of Workplace Flextime Use and Perceived Control. *J. Bus. Psychol.* 36 155–172. 10.1007/s10869-019-09673-4

[B44] Hu L. T. Bentler P. M. (1999). Cutoff Criteria for Fit Indexes in Covariance Structure Analysis: Conventional Criteria Versus New Alternatives. *Struct. Equ. Modeling.* 6 1–55. 10.1080/10705519909540118

[B45] Ji D. Y. Yue Y. P. (2020). Relationship Between Kindergarten Organizational Climate and Teacher Burnout: Work-Family Conflict as a Mediator. *Front. Psychiatry* 11:408. 10.3389/fpsyt.2020.00408 PMC7242754 32499727

[B46] Kwok S. Cheng L. Wong D. F. K. (2015). Family Emotional Support, Positive Psychological Capital and Job Satisfaction Among Chinese White-Collar Workers. *J. Happ. Stud.* 16 561–582.

[B47] Lambert E. G. Minor K. I. Wells J. B. Hogan N. L. (2016). Social support’s relationship to correctional staff job stress, job involvement, job satisfaction, and organizational commitment. *Soc. Sci. J.* 53 22–32. 10.1016/j.soscij.2015.10.001

[B48] Lawson K. M. Davis K. D. Crouter A. C. O’Neill J. W. (2013). Understanding work-family spillover in hotel managers. *Int. J. Hosp.Manag.* 33 273–281. 10.1016/j.ijhm.2012.09.003 23888092 PMC3718488

[B49] Lee Siew Kim J. Seow Ling C. (2001). Work-family conflict of women entrepreneurs in Singapore. *Wom. Manag. Rev*. 16, 204–221. 10.1108/09649420110395692

[B50] Le H. Newman A. Menzies J. Zheng C. Fermelis J. (2020). Work-life balance in Asia: A systematic review. *Hum. Resour. Manage. Rev.* 30:100766. 10.1016/j.hrmr.2020.100766

[B51] Li Z. Lin Y. Fang W. (2015). Social support and job satisfaction: Elaborating the mediating role of work-family interface. *Curr. Psychol.* 34 1–15.

[B52] Liu X. X. Keller J. Hong Y. Y. (2015). Hiring of Personal Ties: A Cultural Consensus Analysis of China and the United States. *Manag. Organ. Rev.* 11 145–169. 10.1017/mor.2015.1

[B53] Luo Y. D. Huang Y. Wang S. L. (2012). Guanxi and Organizational Performance: A Meta-Analysis.Manag. *Organ. Rev.* 8 139–172. 10.1111/j.1740-8784.2011.00273.x

[B54] Marks S. R. (1977). Multiple roles and role strain: Some notes on human energy, time and commitment. *Am. Soc. Rev.* 42 921–936. 10.2307/2094577

[B55] Martin J. A. Buffardi L. C. Erdwins C. J. (2002). Work-family conflict, perceived organization support, and organizational commitment among employed mothers. *J. Occup. Health Psychol.* 7:99. 12003369 10.1037//1076-8998.7.2.99

[B56] Matthews R. A. Barnes-Farrell J. L. (2010). Development and Initial Evaluation of an Enhanced Measure of Boundary Flexibility for the Work and Family Domains. *J. Occup. Health Psychol.* 15 330–346. 10.1037/a0019302 20604638

[B57] Mauno S. Rantanen M. (2013). Contextual and dispositional coping resources as predictors of work–family conflict and enrichment: which of these resources or their combinations are the most beneficial? *J. Fam. Econ.* 34 87–104. 10.1007/s10834-012-9306-3

[B58] McNall L. A. Masuda A. D. Shanock L. R. Nicklin J. M. (2011). Interaction of core self-evaluations and perceived organizational support on work-to-family enrichment. *J. Psychol.* 145 133–149. 21449248 10.1080/00223980.2010.542506

[B59] McNall L. A. Nicklin J. M. Masuda A. D. (2010). A Meta-Analytic Review of the Consequences Associated with Work-Family Enrichment. *J. Bus. Psychol.* 25 381–396. 10.1007/s10869-009-9141-1

[B60] McNall L. A. Scott L. D. Nicklin J. M. (2014). Do Positive Affectivity and Boundary Preferences Matter for Work-Family Enrichment? A Study of Human Service Workers. *J. Occup. Health Psychol.* 20 93–104. 10.1037/a0038165 25347683

[B61] Michel J. S. Clark M. A. (2013). Investigating the relative importance of individual differences on the work-family interface and the moderating role of boundary preference for segmentation. *Stress Health.* 4 324–336. 10.1002/smi.2474 23148037

[B62] Nasurdin A. M. O’Driscoll M. P. (2012). Work overload, parental demand, perceived organizational support, family support, and work-family conflict among New Zealand and Malaysian academics. *N. Z. J. Psychol.* 41 38–48.

[B63] Nicklin J. M. McNall L. A. (2013). Work-family enrichment, support, and satisfaction: A test of mediation. *Eur. J. Work Organ. Psychol.* 22 67–77. 10.1080/1359432x.2011.616652

[B64] Odle-Dusseau H. N. Britt T. W. Greene-Shortridge T. M. (2012). Organizational Work-Family Resources as Predictors of Job Performance and Attitudes: The Process of Work-Family Conflict and Enrichment. *J. Occup. Health Psychol.* 17 28–40. 10.1037/a0026428 22149204

[B65] Parasuraman S. Greenhaus J. H. Granrose C. S. (1992). Role stressors, social support and well-being among two-career couples. *J. Organ. Behav.* 13 339–356. 10.1002/job.4030130403

[B66] Voydanoff P. (2005). The differential aalience of family and community demands and resources for family-to-work conflict and aacilitation. *J. Fam. Econ.Iss.* 26 395–417. 10.1007/s10834-005-5904-7

[B67] Podsakoff P. M. MacKenzie S. B. Lee J. Y. Podsakoff N. P. (2003). Common method biases in behavioral research: A critical review of the literature and recommended remedies. *J. Appl. Psychol.* 88 879–903. 14516251 10.1037/0021-9010.88.5.879

[B68] Powell G. N. (2006). When work and family are allies: a theory of work-family enrichment. *Acad. Manage. Rev.* 31 72–92.

[B69] Powell G. N. Francesco A. M. Ling Y. (2009). Toward culture-sensitive theories of the work-family interface. *J. Organ. Behav.* 30 597–616. 10.1002/job.568

[B70] Powell G. N. Greenhaus J. H. (2006). Managing incidents of work-family conflict: A decision-making perspective. *Hum. Relat.* 59 1179–1212. 10.1177/0018726706069765

[B71] Rastogi M. Rangnekar S. Rastogi R. (2016). Flexibility as a Predictor of Work–Family Enrichment. *Glob. J. Flex. Syst. Manag.* 17 5–14. 10.1007/s40171-015-0108-y

[B72] Rhoades L. Eisenberger R. (2002). Perceived organizational support: A review of the literature. *J. Appl. Psychol.* 87 698–714. 10.1037//0021-9010.87.4.698 12184574

[B73] Rothbard N. P. (2001). Enriching or depleting? The dynamics of engagement in work and family roles. *Adm. Sci. Q.* 46 655–684. 10.2307/3094827

[B74] Siu O. L. Bakker A. B. Brough P. Lu C. Q. Wang H. J. Kalliath T. (2015). A Three-wave Study of Antecedents of Work-Family Enrichment: The Roles of Social Resources and Affect. *Stress Health.* 31 306–314. 10.1002/smi.2556 26468889

[B75] Sonnentag S. Binnewies C. Mojza E. J. (2010). Staying Well and Engaged When Demands Are High: The Role of Psychological Detachment. *J. Appl. Psychol.* 95 965–976. 10.1037/a0020032 20718528

[B76] Staines L. (1980). Spillover versus compensation: a review of the literature on the relationship between work and non-work. *Hum.Relat.* 33 111–129.

[B77] Tang S. W. Siu O. L. Cheung F. (2014). A Study of Work-Family Enrichment among Chinese Employees: The Mediating Role between Work Support and Job Satisfaction. *Appl. Psychol. Int. Rev. Psychol.* 63 130–150. 10.1111/j.1464-0597.2012.00519.x

[B78] Tement S. Korunka C. (2013). Does Trait Affectivity Predict Work-to-Family Conflict and Enrichment Beyond Job Characteristics?. *J. Psychol.* 147 197–216. 10.1080/00223980.2012.683053 23469478

[B79] ten Brummelhuis L. L. Bakker A. B. (2012). A Resource Perspective on the Work-Home Interface The Work-Home Resources Model. *Am. Psychol.* 67 545–556. 10.1037/a0027974 22506688

[B80] Voydanoff P. (2004). The effects of work demands and resources on work-to-family conflict and facilitation. *J. Marr. Fam.* 66 398–412. 10.1111/j.1741-3737.2004.00028.x

[B81] Wattoo M. A. Zhao S. M. Xi M. (2018). Perceived organizational support and employee well-being: Testing the mediatory role of work-family facilitation and work-family conflict.Chin. *Manag. Stud.* 12 469–484.

[B82] Wayne J. (2009). “Reducing conceptual confusion: clarifying the positive side of work and family” in *Handbook of Families and Work: interdisciplinary Perspectives.* eds Crane D. R. Hill J. (Lanham: University Press of America). 105–140.

[B83] Wayne J. H. Grzywacz J. G. Carlson D. S. Kacmar K. M. (2007). Work–family facilitation: A theoretical explanation and model of primary antecedents and consequences. *Hum. Resour. Manag. Rev.* 17 63–76. 10.1016/j.hrmr.2007.01.002

[B84] Wayne J. H. Randel A. E. Stevens J. (2006). The role of identity and work-family support in work-family enrichment and its work-related consequences. *J. Vocat. Behav.* 69 445–461. 10.1016/j.jvb.2006.07.002

[B85] Westman M. Brough P. Kalliath T. (2009). Expert commentary on work-life balance and crossover of emotions and experiences: theoretical and practice advancements. *J. Organ. Behav*. 30, 587–595. 10.1002/job.616

[B86] Zhang H. N. Kwan H. K. Everett A. M. Jian Z. Q. (2012). Servant leadership, organizational identification, and work-to-family enrichment: The moderating role of work climate for sharing family concerns. *Hum. Resour. Manag.* 51 747–767. 10.1002/hrm.21498

[B87] Zhang L. Lin Y. C. Wan F. (2015). Social Support and Job Satisfaction: Elaborating the Mediating Role of Work-Family Interface. *Curr. Psychol.* 34 781–790. 10.1007/s12144-014-9290-x

[B88] Zhang L. M. Yu S. L. Jiang L. J. (2020). Chinese preschool teachers’ emotional labor and regulation strategies. *Teach. Teach. Educ.* 92:103024. 10.1016/j.tate.2020.103024

[B89] Zhang L. M. Yu S. L. Liu H. (2019). Understanding teachers’ motivation for and commitment to teaching: profiles of Chinese early career, early childhood teachers. *Teach. Teach.* 25 890–914. 10.1080/13540602.2019.1670155

[B90] Zhou S. Y. Li X. W. Gao B. C. (2020). Family/friends support, work-family conflict, organizational commitment, and turnover intention in young preschool teachers in China: A serial mediation model. *Child. Youth Serv.Rev.* 113:104997. 10.1016/j.childyouth.2020.104997

